# Multiplex Polymerase Chain Reaction Panels for Gastrointestinal Infections: Current Evidence, Regulatory Hurdles, and the Way Forward

**DOI:** 10.1093/ofid/ofaf445

**Published:** 2025-10-22

**Authors:** Giannoula S Tansarli, David R Allen, Ferric C Fang

**Affiliations:** Department of Laboratory Medicine and Pathology, School of Medicine, University of Washington, Seattle, Washington, USA; US Medical Affairs, bioMérieux, Salt Lake City, Utah, USA; Department of Laboratory Medicine and Pathology, School of Medicine, University of Washington, Seattle, Washington, USA; Department of Microbiology, School of Medicine, University of Washington, Seattle, Washington, USA

**Keywords:** diarrhea, gastroenteritis, molecular diagnostics, reimbursement, syndromic testing

## Abstract

Syndromic multiplex polymerase chain reaction (PCR) panels have revolutionized the diagnosis of gastrointestinal infections, allowing the rapid and simultaneous detection of multiple pathogens, including rare or difficult-to-identify organisms, with superior analytic sensitivity as compared with conventional methods. Although multiplex PCR panels are costly, their costs are offset by lower health care costs resulting from improved diagnostic accuracy and more targeted therapy. However, significant barriers to reimbursement may discourage providers from ordering PCR panels or incentivize them to use smaller panels that are less comprehensive. Addressing these challenges will require a collaborative effort, including regulators, payors, and clinicians. Key steps will include updating clinical guidelines to better define appropriate utilization of gastrointestinal panels, harmonizing reimbursement criteria to align with evidence-based practice, and modernizing diagnostic codes for acute gastroenteritis to match payors’ requirements. These reforms will be essential to improve access to advanced diagnostics and ensure better patient care.

## EPIDEMIOLOGY OF ACUTE GASTROENTERITIS IN THE UNITED STATES

Acute gastroenteritis remains one of the most frequent reasons for urgent care and outpatient clinic visits in the United States [1] with an estimated 179 million cases per year and a health care cost burden exceeding $300 million annually in adults alone [2]. Among adults, females are affected more frequently than males [2], and young children, particularly those in daycare settings, are disproportionately affected as compared with adults [3]. Most cases are caused by viruses and result in self-limiting diarrhea, with or without vomiting, with norovirus being the leading culprit due to its highly contagious nature [4–6]. Prior to the availability of rotavirus vaccines for infants, rotavirus was the most common cause of severe gastroenteritis in children, sometimes requiring hospitalization [7]. Despite the effectiveness of the rotavirus vaccine, infant vaccination rates in the United States have plateaued <80%, allowing rotavirus to remain a significant cause of illness [8, 9]. Although most cases of gastroenteritis are viral, ruling out bacterial and parasitic infections is essential for public health management and to determine which patients might benefit from antimicrobial treatment.

Bacterial and parasitic causes of diarrhea are comparatively less common in high-income countries (HICs) but pose significant health burdens in vulnerable populations. These infections are predominantly transmitted through contaminated food or water and are of particular concern from a public health perspective [1]. In the United States, cases are most frequently observed among returning travelers from low- and middle-income countries (LMICs) or as part of domestic outbreaks [10, 11]. *Campylobacter* is the leading foodborne bacterial cause, followed by *Escherichia coli*, *Salmonella*, and *Shigella* [1]. Bacterial and parasitic infections are often more severe than viral gastroenteritis and are characterized by significant inflammation, with some cases progressing to dysentery [1].

Certain transmission routes render specific populations more vulnerable to acute gastrointestinal infections. For example, fecal-oral transmission is associated with elevated rates of infection with a range of pathogens in men who have sex with men (MSM) [12, 13] and in persons experiencing homelessness (PEH) [14, 15]. Individuals who are immunocompromised are at increased risk for gastrointestinal infections, with some populations being particularly vulnerable. Individuals with HIV are more susceptible to cryptosporidiosis, while patients with hematologic malignancies are prone to viral gastroenteritis [16]. In these cases, the immunocompromised state often leads to prolonged and severe symptoms, making the rapid identification of the causative organism essential to ensure timely and appropriate therapy.

## DIAGNOSTIC TESTS FOR GASTROENTERITIS

The laboratory diagnosis of infectious gastroenteritis involves a range of testing methods. Historically, bacterial culture has been used to identify *Campylobacter*, *Salmonella*, *Shigella*, and Shiga toxin–producing *E coli* (eg, serotype O157:H7) [17]. Bacterial culture allows for antibiotic susceptibility testing when needed and for strain typing, which is essential when epidemiologic investigations need to be performed. However, cultures exhibit variable sensitivity, with some organisms exhibiting low detection rates, and require a turnaround time of 2 to 3 days. Routine laboratory testing is not necessary for the diagnosis of viral gastroenteritis, as it is typically mild and self-limiting. Antigen-based tests for certain viruses were once used but have been largely abandoned due to their low sensitivity and specificity. For parasitic infections, microscopic examination for ova and parasites was once considered the gold standard, but its sensitivity is limited, often requiring the collection of multiple samples on different days to improve yield and experienced technologists to perform [18].

The limitations of conventional testing have driven the development of syndromic multiplex polymerase chain reaction (PCR) panels, which simultaneously test for the presence of multiple pathogens [19]. These nucleic acid amplification tests (NAATs) aim to identify the most common bacteria, viruses, and parasites that cause community-acquired gastroenteritis. Since the first multiplex PCR panel for stool samples became available in the United States in 2015, these panels have been widely adopted and are now the cornerstone of laboratory diagnostics for infectious diarrhea.

Although the role of bacterial stool culture is limited, culture is still necessary for public health surveillance [20], susceptibility testing, and the recovery of emerging enteric pathogens that are not included in panels (eg, *Aeromonas* spp, unusual *Campylobacter* spp, *Providencia alcalifaciens*, *Escherichia albertii*, *Klebsiella oxytoca*, *Laribacter hongkongensis*) [17]. Serologic typing or whole genome sequencing of *Shigella*, *Salmonella*, *Campylobacter*, or Shiga toxin–producing *E coli* requires cultured isolates and is performed by public health laboratories. Positive PCR detections of these organisms are therefore generally reflexively set up for culture, along with susceptibility testing as appropriate.

Several commercially available NAAT platforms are now in use across the United States. These include the BioFire FilmArray system [21] and the recently launched BioFire FilmArray GI Panel Mid with fewer targets, the xTag GI pathogen panel [22–26], the Verigene enteric pathogens panel [27], the QIAstat-Dx GIP [28], the BioCode GPP [29], and various panels offered for the BD MAX system [30–34]. The organisms covered by these platforms are presented in Table 1.

**Table 1.** Multiplex Polymerase Chain Reaction Gastrointestinal Panels Available in the United States

Panel: Target Menu			
BioFire FilmArray GIP	BioFire FilmArray GIP Mid	BD MAX Assays	xTAG GPP
*Campylobacter* (*C jejuni*, *C coli*, *C upsaliensis*) *C difficile* (toxin A/B) *Plesiomonas shigelloides* *Salmonella* *Yersinia enterocolitica* *Vibrio* (*V parahaemolyticus*, *V vulnificus*, *V cholerae*) EAEC EPEC ETEC *lt*/*st* STEC *stx1*/*stx2* *Shigella*/EIEC Adenovirus F40/41 Astrovirus Norovirus GI/GII Rotavirus A Sapovirus (I, II, IV, V) *Cryptosporidium* *Cyclospora cayetanensis* *Entamoeba histolytica* *Giardia duodenalis*	*Campylobacter* (*C jejuni*, *C coli*, *C upsaliensis*) *C difficile* (toxin A/B) *Salmonella* *Yersinia enterocolitica* *Vibrio* (*V parahaemolyticus*, *V vulnificus*, *V cholerae*) STEC *stx1*/*stx2* *Shigella*/EIEC Norovirus GI/GII *Cryptosporidium* *Cyclospora cayetanensis* *Giardia duodenalis*	**EBP** *Salmonella* spp *Campylobacter* spp (*C jejuni*, *C coli*) *Shigella* spp/EIEC Shiga toxin 1/2 **Extended EBP** *Plesiomonas shigelloides* *Vibrio* (*V vulnificus*, *V parahaemolyticus*, *V cholerae*) ETEC heat-labile/heat-stable enterotoxin genes *Yersinia enterocolitica* **EVP** Norovirus GI/GII Rotavirus A Adenovirus F40/41 Sapovirus (I, II, IV, V) Human astrovirus (hAstro) **EPP** *Giardia duodenalis* *Cryptosporidium* *Entamoeba histolytica* **Cdiff** *C difficile* toxin B gene (*tcdB*)	*Campylobacter* *C difficile*, toxin A/B *E coli* O157 ETEC LT/ST STEC *stx1/stx2* *Salmonella* *Shigella* *Vibrio cholerae* Adenovirus 40/41 Norovirus GI/GII Rotavirus A *Cryptosporidium* *Giardia* *Entamoeba histolytica*

Verigene EP	QIAstat-Dx GIP	BioCode GPP
*Campylobacter* group *Salmonella* spp *Shigella* spp *Vibrio* group *Yersinia enterocolitica* Shiga toxin 1 (*stx1*) Shiga toxin 2 (*stx2*) Norovirus (GI/GII) Rotavirus (group A)	*Clostridium difficile* toxin A/B EAEC EPEC ETEC LT/ST STEC *stx1*/*stx2* STEC O157:H7 EIEC/*Shigella* *Campylobacter* spp (*C jejuni*, *C upsaliensis*, *C coli*) *Plesiomonas shigelloides* *Salmonella* spp *Vibrio* spp (*V cholerae, V parahaemolyticus*, *V vulnificus*) *Yersinia enterocolitica* *Cyclospora cayetanensis* *Cryptosporidium* spp *Entamoeba histolytica* *Giardia duodenalis* Adenovirus F40/41 Astrovirus Norovirus GI/GII Rotavirus Sapovirus (I, II, IV, V) **QIAstat-Dx GIP 2 Mini B&V** *Campylobacter* *Salmonella* STEC *Shigella* Norovirus	*Campylobacter* (*C jejuni*, *C coli*) *C difficile* toxin A/B *Salmonella* spp STEC *stx1*/*stx2* *Shigella*/EIEC *E coli* O157 ETEC LT/ST EAEC *Vibrio* spp (*V cholerae*, *V parahaemolyticus*, *V vulnificus*) *Yersinia enterocolitica* Adenovirus F 40/41 Norovirus GI/GII Rotavirus A *Cryptosporidium* *Giardia duodenalis* *Entamoeba histolytica*

Abbreviations: *C difficile*, *Clostridioides difficile*; EAEC, enteroaggregative *E coli*; EBP, enteric bacterial panel; *E coli*, *Escherichia coli*; EIEC, enteroinvasive *E coli*; EP, enteric pathogens; EPEC, enteropathogenic *E coli*; EPP, enteric parasite panel; ETEC, enterotoxigenic *E coli*; EVP, enteric viral panel; GIP, gastrointestinal panel; GPP, gastrointestinal pathogen panel; STEC, Shiga toxin–producing *E coli*.

While PCR panels are analytically highly sensitive, they are not without limitations. One key issue is their inability to distinguish between active infection and asymptomatic colonization, which is common for many potential pathogens, such as *Clostridioides difficile* [35], and poses particular challenges in immunocompromised populations at high risk for noninfectious gastrointestinal disease, such as hematopoietic stem cell transplant recipients [36]. Moreover, the clinical significance of some potential enteric pathogens, such as enteropathogenic and enteroaggregative *E coli* (EPEC and EAEC), in otherwise healthy adults with diarrhea remains to be established [37, 38]. Another limitation is that PCR panels do not detect all foodborne pathogens (eg, *Listeria monocytogenes*) or certain toxin-mediated foodborne illnesses, such as those caused by *Staphylococcus aureus*, *Bacillus cereus*, *Clostridium botulinum*, or *Clostridium perfringens*, which account for a substantial number of episodes of acute gastrointestinal disease [39] and require specialized testing by public health laboratories. With the exception of *Giardia*, *Entamoeba histolytica*, *Cryptosporidium*, and *Cyclospora*, parasitic causes of diarrhea, such as microsporidia, *Strongyloides*, and *Ascaris*, are not detected by PCR panels.

## CLINICAL IMPACT

Multiplex PCR panels for gastrointestinal infections exhibit high analytic sensitivity (94.5%–100%) and analytic specificity (≥96.5%) [21, 24, 40–42]. Unlike traditional methods, these panels can identify pathogens that are often overlooked or untested, such as diarrheagenic *E coli*, *Plesiomonas shigelloides*, and certain viruses. Multiplex PCR panels detect 3 to 5 times more pathogens than other diagnostic methods used for patients with community-acquired gastroenteritis [43, 44]. In addition, multiplex panels identify mixed infections more frequently than do conventional diagnostic methods [24, 43, 45]. Another advantage of large syndromic panels is the reduced need for health care providers to navigate complex order sets or stepwise algorithms for bacteriology and parasitology testing that may cause significant delays in care [46].

The rapid turnaround time of multiplex PCR panels is advantageous from a public health standpoint and facilitates the treatment of patients with highly infectious causes of diarrhea (eg, shigellosis) in whom the prompt initiation of antibiotic therapy can mitigate community transmission [15]. Conversely, a negative result from a panel can reduce unnecessary antibiotic use [47–51] and promote the more rapid initiation of targeted (ie, diagnostic result-based) therapy [44, 50, 51]. This is particularly important in Shiga-like toxin–producing *E coli* (STEC) infections, where the rapid identification of the organism can lead to immediate withholding or discontinuation of empirical antibiotics to avert hemolytic uremic syndrome [44]. While the ability to rapidly detect STEC is of great clinical importance, the high sensitivity of PCR has been shown to significantly improve the rate of STEC detection, with one study demonstrating a 45% increase in detections by PCR as compared with a Shiga toxin enzyme immunoassay (EIA) and a nearly 38% increase in detections as compared with culture [8]. Numerous studies have shown that multiplex panels are associated with turnaround times of hours vs days [44, 52, 53] and 3- to 5-fold higher pathogen detection rates [44, 52–56] than conventional testing. This improved diagnostic yield paired with expedited turnaround time is thought to lead to a range of potential downstream clinical and economic benefits. For instance, 2 studies found that multiplex panels are associated with shorter hospital length of stay [47, 51], 1 of which also demonstrated a cost savings of >$293 per patient [47]. Three additional studies observed a reduction in the need for imaging studies or endoscopies in patients tested with multiplex panels [47, 49, 54, 57]. The impact on infection control was evaluated in 3 studies, which found that multiplex panels are associated with more appropriate implementation of isolation measures in the hospital, enabling faster initiation or discontinuation of isolation precautions, with the potential for significant cost savings [49, 58, 59].

Several studies have assessed the impact of multiplex panels in specific patient populations. Multiplex panels have proven especially valuable in patients who are immunosuppressed, with one study in hematopoietic cell transplant recipients observing an increased detection rate of non–*C difficile* infectious etiologies ranging from 5% to 27% after implementation of a multiplex PCR panel, without an increase in overall testing costs [60]. In a study of patients who were immunosuppressed and tested with a gastrointestinal panel, researchers found that the results affected antimicrobial management in >50% of cases [61]. Patients with inflammatory bowel disease (IBD) are often immunosuppressed and benefit when PCR panels are able to exclude infectious causes during a disease flare. An association between infection and IBD flares was demonstrated in one study, which found 31.1% of IBD cases to have a potential pathogen detected by PCR during a symptomatic flare as opposed to only 7.6% of those with quiescent disease [62]. Investigators in this and 2 additional studies found that detection of a potential pathogen is associated with lower rates of IBD treatment modification [57, 62, 63] and fewer endoscopies [57, 63]. Studies evaluating the use of multiplex panels in the emergency department have reported higher rates of detection of clinically relevant pathogens [64], fewer return visits [64], decreased patient admissions [65], reduced overall patient charges [65], and tailoring of therapy based on the organisms detected [10, 66, 67].

Notably, multiplex PCR panels have detected an unexpectedly high prevalence of diarrheagenic *E coli* in HICs, including EPEC, EAEC, and enterotoxigenic *E coli* [65, 68–70]. These *E coli* detections may provide an otherwise unrecognized cause of illness but can also lead to challenges with interpreting the clinical significance of these results. In addition, PCR panels have facilitated the unsuspected detection of outbreaks caused by *Cyclospora cayetanensis* and *Cryptosporidium* spp, parasites traditionally identified by acid-fast staining of stool samples [71], and *Shigella* spp [72, 73]. Several studies have highlighted the utility of multiplex panels in rapidly detecting *Shigella*, *Campylobacter*, diarrheagenic *E coli*, *Giardia*, and *Cryptosporidium* among MSM who are at increased risk for sexually transmitted enteric pathogens, thereby facilitating the public health response [13, 15, 74, 75]. Outbreaks of shigellosis have also been identified among individuals with poor access to hygiene in HICs, particularly among PEH. In such cases, the use of multiplex PCR panels has been instrumental in the rapid recognition and containment of infections, preventing further spread within the community [15]. The ability of multiplex panels to simultaneously detect multiple pathogens has shown that mixed infections primarily occur in specific at-risk populations: MSM, travelers returning from LMICs, and PEH [44, 75, 76].

Smaller diagnostic panels that detect a limited number of organisms have been developed and may appeal to some health care providers due to lower reagent costs. A recent study evaluated health care outcomes and costs associated with large multiplex panels (≥12 targets), smaller multiplex panels (<12 targets), or conventional testing in adult outpatients presenting to >1000 US hospitals with acute infectious gastroenteritis over a 5-year period [77]. Although the use of multiplex PCR panels with ≥12 targets was associated with slightly higher initial health care costs, they were associated with positive outcomes, including a higher percentage of patients being discharged home, less secondary testing and diagnostic imaging, and fewer hospitalizations during the subsequent 30 days. As a result, combined health care costs for the initial visit and 30-day follow-up period were comparable in patients tested with large panels and those tested by traditional stool testing, and combined costs associated with smaller panels were the highest. The use of larger multiplex PCR panels was also associated with decreased in-hospital administration of antibiotics, suggesting improvements in antimicrobial stewardship [77]; this most likely represents a benefit of the detection of viral pathogens in the absence of bacterial pathogens, which allows clinicians to withhold antibiotics with greater confidence. This effect was similarly observed in a multicenter prospective study conducted across 5 US academic children's hospitals [64]. That study compared a large multiplex PCR panel with clinician-selected testing in the emergency department and found a 21% overall reduction in the odds of a return visit in the multiplex panel group, with an even more substantial reduction of 54% when a viral pathogen was identified [64].

### Diagnostic Stewardship Considerations

Acute gastroenteritis is typically a self-limiting condition that does not require laboratory testing or antibiotic treatment. Diagnostic evaluation is warranted in certain situations: for patients with prolonged or severe symptoms, during outbreak investigations, for individuals who are immunocompromised and at greater risk of complications, and for those who pose a high risk of transmitting the disease to others (eg, day care workers, food handlers). Despite the well-documented advantages of multiplex PCR panels, establishing clear consensus testing criteria is important to prevent unnecessary use. Stool diagnostic studies were recommended in the 2016 clinical guideline of the American College of Gastroenterology in patients presenting with dysentery, moderate to severe illness, or symptoms persisting for >7 days, to identify the underlying cause and facilitate tailored treatment [78]. The 2017 guidelines of the Infectious Diseases Society of America (IDSA) advocate diagnostic testing in patients presenting with symptoms of fever, abdominal pain, or blood in the stool and in those with immunocompromising conditions [79]. In addition, multiplex panels are usually not indicated in patients with diarrhea who have been hospitalized for >3 days, in whom targeted *C difficile* PCR testing is recommended instead [50, 80].

Diagnostic stewardship refers to the use of laboratory testing to guide patient management to optimize clinical outcomes and limit the spread of antimicrobial resistance [81]. Diagnostic stewardship has a complementary relationship with antimicrobial stewardship and can be viewed as implementing diagnostic testing that is appropriate for the clinical setting and in appropriate patients [82]. By providing rapid and accurate diagnostic information, multiplex PCR testing can allow more targeted management of patients with acute gastroenteritis [44] and help to fulfill the goals of diagnostic stewardship [15].

Like other sensitive molecular diagnostic assays, gastrointestinal multiplex PCR tests require attention to diagnostic stewardship principles across all phases (eg, preanalytic, analytic, postanalytic) to optimize their use and subsequent impact on patient care [83]. In the preanalytic phase, several important considerations have been noted as key to reducing test overuse, including not testing stool from patients taking laxatives or from patients who have been admitted to the hospital for ≥3 days [50, 80]. Additionally, patients should meet clinical criteria at the time of specimen collection, which includes diarrhea (particularly if inflammatory, severe, or lasting >7 days), known exposure to an outbreak caused by a gastrointestinal pathogen, travel history to areas with endemic gastrointestinal pathogens, or immunocompromised status [50, 80]. Several studies have demonstrated that addressing these preanalytic indicators improves pretest probability and decreases excessive test use [50, 53, 80, 84]. In the analytic phase, some laboratories have chosen to selectively report some results, such as *C difficile* [85], but this practice has been associated with missed diagnoses of *C difficile* infection (CDI) in patients lacking traditional risk factors [86] (discussed later). An exception is made for infants and young children, who have high rates of asymptomatic *C difficile* carriage; routine reporting of *C difficile* detection in children less than 1 to 2 years of age is therefore discouraged [87]. Similarly, some laboratories do not report detection of enteropathogenic or enteroaggregative *E coli* because they may represent asymptomatic colonization or self-limited infections [84]. However, these organisms can be responsible for persistent symptoms that benefit from specific treatment [69, 88–90]. As with *C difficile*, clinicians must be informed that diarrheagenic *E coli* strains are present in order to consider the clinical significance in individual patients. Laboratories may also take steps to ensure specimen appropriateness prior to testing, such as rejecting formed stools that do not take the shape of the container [18, 91]. Overall, diagnostic stewardship can minimize unnecessary testing and optimize the clinical impact of multiplex PCR panels [79, 84, 92–94].

### Current Regulatory Barriers and Reporting Issues

Despite the demonstrated clinical benefits and cost-effectiveness of syndromic testing for gastrointestinal infections [44, 47, 51, 64, 77], significant reimbursement challenges remain, with many payors reluctant to cover “expanded” panels (defined as either >5 or >11 targets). This issue is closely tied to the existence of multiple local coverage determinations (LCDs), including L38229, L38988, L39003, and L39044 [95–98], which were issued by Medicare Administrative Contractors (MACs). A summary of these LCDs is presented in Table 2. LCDs specifically apply to Medicare patients, but private payors often model their coverage policies on LCDs, giving them broad relevance in shaping reimbursement criteria. Billing and coding articles, such as A58710, A58726, A58720, and A58963 [99–102], accompany the relevant LCDs and provide detailed instructions for claim submission. These articles explain appropriate procedure codes, diagnosis codes, and modifiers required for reimbursement. Together, the LCDs and their associated billing and coding articles create a complex framework for reimbursement, posing challenges for providers navigating varied regional policies, clinical use criteria, and payor policies.

**Table 2.** LCDs on the Insurance Coverage of Multiplex Polymerase Chain Reaction Panels With ≥12 Targets

MolDX	Criteria for Reimbursement
L37364 LCD: Foodborne GI panels identified by multiplex NAATs (CGS administrators)	Limit GI panel coverage in immune-competent beneficiaries up to 5 bacterial targets.
L37709 LCD: Foodborne GI panels identified by multiplex NAATs (Palmetto)	Testing for ≥12 organisms will be covered only in patients who are critically ill or immunosuppressed. GIP testing is limited to no more than 5 bacterial pathogen targets when not testing for *C difficile*; testing for 6–11 pathogens is covered when there is a clinical concern for *C difficile*. When community-acquired diarrhea persists for ≥7 d, or the diarrhea is travel related, or there are signs/symptoms of severe disease, GIP testing may be warranted.
L38229 LCD: GI pathogen panels utilizing multiplex nucleic acid amplification techniques (Novitas)	GI panels with ≤11 targets are medically reasonable and necessary for the evaluation of Medicare beneficiaries with the following: Acute diarrhea at least 7 d Persistent diarrhea of 14–30 d Fever, blood, dysentery, dehydration, severe abdominal pain, hospitalization, or immunocompromise. ≥12 targets for immunocompromise and acute or persistent diarrhea.
L39038 LCD: MolDX—molecular syndromic panels for ID pathogen identification testing (CGS)	Limited coverage for expanded (>5 pathogens) panel testing GI panels For immune-competent patients at least 1 of the following must apply: Ordered by a specialist in ID or gastroenterology for a patient with severe and established GI pathology Patient is seriously or critically ill or at imminent risk Clinical indication is acute or persistent diarrhea with signs/risk factors for severe disease and/or not resolving after 7 d For patients who are immune suppressed: ordered by an ID, GI, oncologist, transplant or critical care specialist.
L39003 LCD: MolDX—molecular syndromic panels for ID pathogen identification testing (Noridian)	The selected cutoff is <6 pathogens for limited panels and ≥6 for expanded panels. Limited coverage for expanded (>5 pathogens) panel testing GI panels will be covered according to only the following criteria. For patients who are immune competent, at least 1 of the following must apply: Ordered by a specialist in ID or gastroenterology for a patient with severe and established GI pathology Patient is seriously or critically ill or at imminent risk Clinical indication is acute or persistent diarrhea with signs/risk factors for severe disease and/or not resolving after 7 d For patients who are immune-suppressed: ordered by an ID, GI, oncologist, transplant or critical care specialist.
L38988 LCD: MolDX—molecular syndromic panels for ID pathogen identification testing (CGS)	GI panels For patients who are immune competent, at least 1 of the following must apply: Ordered by a specialist in ID or gastroenterology for a patient with severe and established GI pathology Patient is seriously or critically ill or at imminent risk Clinical indication is acute or persistent diarrhea with signs/risk factors for severe disease and/or not resolving after 7 d For patients who are immune suppressed: ordered by an ID, GI, oncologist, transplant or critical care specialist.

Billing and Coding	Article Guidance
A58710	For expanded (>5 pathogens) GIP, the following additional conditions apply: Testing is billed according to 1 of the following: POS^a^ 19, 21, 22, 23, or The test is ordered as follows (for health care POS other than the POS listed in 1[a]): (1) For immune-competent beneficiaries, the test must be ordered by an ID specialist or gastroenterologist who is diagnosing and treating the beneficiary. (2) For immune-compromised beneficiaries, the test must be ordered by a clinician specialist in 1 of the following: ID, oncologist, transplant, or GI who is diagnosing and treating the beneficiary. (3) Regarding (1) and (2), An exception may be made in geographic locations where the specialist cannot be reasonably reached by the beneficiary, and the ordering provider is located closer to the beneficiary's place of residence than the nearest specialist. We would generally expect that beneficiaries for whom the test is ordered under this exception to be living in rural locations, islands, or some other location where access to care is limited. (4) An *ICD-10* diagnosis code from group 7 must be on the claim, in addition to the sign or symptom from group 2 for which there is suspicion of GI illness to bill for the GIP. The exception to this is testing that is performed as part of a pretransplant evaluation of an immune-compromised beneficiary, regardless of the presence of symptoms. In such cases, clear documentation of the pretransplant evaluation must accompany the claim. The expanded/targeted panel distinction is not applicable to all panels, except as otherwise indicated in the related policy.
A58726	Same as is A58710
A58720	Same as in A58710 and A58726
A58963	Billing and coding article specific to AGE *Coding information* Procedure codes may be subject to NCCI edits or hospital OPPS packaging edits. Refer to NCCI and OPPS requirements prior to billing Medicare. For services requiring a referring/ordering physician, the name and NPI of the referring/ordering physician must be reported on the claim. A claim submitted without a valid *ICD-10-CM* diagnosis code will be returned to the provider as an incomplete claim under section 1833(e) of the Social Security Act. The diagnosis codes must best describe the patient's condition for which the service was performed. For diagnostic tests, report the result of the test if known; otherwise, the symptoms prompting the performance of the test should be reported. *Specific coding information (highly multiplexed GPPs: group 2 CPT codes)* Must be billed in POS 20, 21, 22, 23, or 81 (urgent care, inpatient hospital, outpatient hospital [observation], or emergency room) or in POS 81 (independent laboratory) in the case of a pretransplant evaluation for an immune-compromised beneficiary. Outside of one of these POS, the test must be ordered by a clinician specialist in one of the following: ID (44), gastroenterology (10), oncology (839091), or transplant who is diagnosing and treating the beneficiary.* The specialist communication must be documented in the medical record. *An exception may be made in geographic locations where neither specialist can be reasonably reached by the beneficiary and the ordering provider is located closer to the beneficiary's place of residence than the nearest ID specialist. We would generally expect that beneficiaries for whom the test is ordered under this exception to be living in rural locations, islands, or some other location where access to care is limited. *Documentation requirements* The patient's medical record must contain documentation that fully supports the medical necessity for services included within the attached LCD. (See “Indications and Limitations of Coverage.”) This documentation includes, but is not limited to, relevant medical history, physical examination, and results of pertinent diagnostic tests or procedures.

Abbreviations: AGE, acute gastroenteritis; *C difficile*, *Clostridioides difficile*; CGS, Celerian Group Company; GI, gastrointestinal; GIP, gastrointestinal panel; GPP, gastrointestinal pathogen panel; ID, infectious diseases; LCD, local coverage determination; MolDX, Molecular Diagnostic Services Program; NAAT, nucleic acid amplification test; NCCI, National Correct Coding Initiative; NPI, National Provider Identifier; OPPS, Outpatient Prospective Payment System; POS, places of service.

^a^Details on POS can be found on the Centers for Medicare and Medicaid Services website at https://www.cms.gov/medicare/coding-billing/place-of-service-codes/code-sets.

For example, the most recent LCD, L39044, specifies that in patients who are immunocompetent, coverage for expanded gastrointestinal panels will be limited. Criteria for coverage include the order being placed by an expert provider, a clear anticipation that the test result will lead to changes in patient management, severe illness caused by the gastrointestinal infection, or diarrhea lasting >7 days [95]. In some cases, these restrictive policies may prompt providers to shift toward smaller panels, potentially leading to the prioritization of cost containment over clinical effectiveness. Reimbursement guidelines may incentivize providers or laboratories to opt for less comprehensive panels that result in suboptimal diagnostic accuracy and missed detection of important pathogens.

Furthermore, the *ICD-10* coding system poses significant challenges for acute gastroenteritis due to its complexity and lack of specificity. Although pathogen-specific codes exist for certain organisms, broad, nonspecific codes are applied when the causative pathogen is unknown, potentially leading to reimbursement denials. *ICD-10* does not provide codes that capture critical factors such as the severity or duration of symptoms, which are often required by payors to justify the use of expanded gastrointestinal panels. This discrepancy forces providers to rely on symptom codes such as R19.7 (diarrhea, unspecified) or R50.9 (fever, unspecified), which are insufficient on their own to convey the clinical necessity of testing and ensure successful reimbursement. These coding challenges become even more complex in cases involving mixed infections or prolonged symptoms. As a result, payor-driven requirements combined with limited coding granularity contribute to inconsistencies and delays in the reimbursement process.

Another issue is the suppression of results for *C difficile*, EPEC, and EAEC by many laboratories performing multiplex PCR testing in the United States. The clinical significance of EPEC and EAEC in HIC patients with diarrhea is presently uncertain, with most evidence coming from studies in pediatric populations in LMICs. In adults, EPEC and EAEC appear to cause a spectrum of clinical presentations, ranging from asymptomatic carriage to mild, self-limited diarrhea, but few studies have focused on these pathogens in adults. In addition, the risk factors for EPEC and EAEC acquisition in HICs are poorly understood. Due to their unclear clinical relevance, EPEC and EAEC are not included in the IDSA guidelines for the treatment of infectious diarrhea [79]. However, numerous reports indicate that EPEC and EAEC can cause persistent gastrointestinal symptoms in adults in HICs, which may lead to adverse health outcomes if left untreated [69, 88–90, 103–105].

Similarly, microbiology laboratories frequently withhold the reporting of *C difficile* results from multiplex PCR panels due to concerns about overdiagnosis [86, 106–108]. This practice stems from the fact that NAATs cannot distinguish between active infection and asymptomatic colonization, leading to concern that the detection of *C difficile* may reflect colonization rather than true CDI. However, a recent meta-analysis of 46 studies found that patients who are NAAT positive/EIA negative have a similar risk of mortality and CDI-related complications as those who are NAAT positive/EIA positive and that the majority of NAAT-positive/EIA-negative cases still recieve CDI therapy [109]. The proportion of CDI occurring in the community setting is rising [110], and community-acquired infections may be unsuspected by clinicians because of milder symptoms and the absence of traditional risk factors [111–114]. *C difficile* was the most common pathogen detected among >12 000 US outpatients from 2016 to 2021 [2]. A recent study reported that suppressing the reporting of *C difficile* results on multiplex panels yielded a 12.6% rate of missed CDI diagnoses, leading to serious complications [86].

### Recommendations

**Figure 1.**
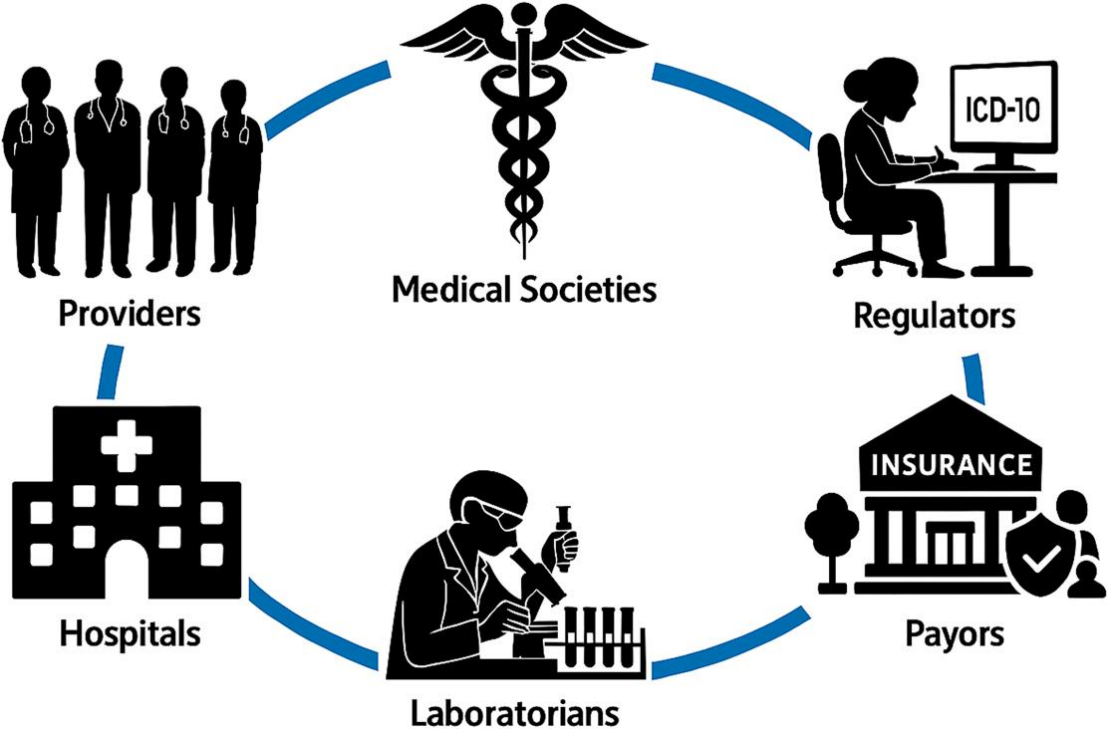
Stakeholders who could contribute to the optimal use of multiplex polymerase chain reaction panels in the diagnosis of acute gastroenteritis.

Several reforms are needed to fully leverage multiplex PCR testing in the diagnosis of acute gastrointestinal infections and allow it to become more consistently accessible to patients. Realization of these reforms will require contributions from multiple stakeholders, including regulators, payors, laboratorians, hospitals, providers, and medical societies (Figure 1). The inconsistencies of LCDs pertaining to gastrointestinal panels should be harmonized with regard to coverage criteria across MACs to promote uniformity in reimbursement policies nationwide. Furthermore, the criteria should avoid arbitrary limits on panel size and should take into account the recent evidence demonstrating that the use of larger panels is associated with improved clinical outcomes [77] (Table 3). A standardized approach should ensure that clinical indications such as prolonged or severe symptoms and high-risk conditions are consistently recognized as justifications for testing, regardless of region. Payors should align LCDs with evolving medical evidence and guidelines to reflect the clinical utility of expanded panels and their ability to detect rare pathogens or mixed infections that can affect patient management. Collaboration among MACs, physicians, and policy makers could also help to standardize reimbursement criteria, reducing the administrative burden on providers. Transparent communication about LCD updates and alignment of billing and coding articles with these policies would further streamline the reimbursement process.

**Table 3.** Relative Ratios of Smaller and Larger Gastrointestinal Panels vs Traditional Stool Workup [77]

	Relative Ratio	
Outcome	Multiplex PCR <12 Targets	Multiplex PCR ≥12 Targets
Cost		
Cost of index visit	1.08	1.04
Cost of 30-d follow-up	0.74	0.74
Cost of index visit + follow-up	1.03	1.00
Clinical outcomes		
In-hospital receipt of antibiotics	1.02	0.84
Likelihood of discharge home	1.20	1.66
Likelihood of 30-d admission	0.74	0.61
Diagnostic outcomes		
Mean number of stool tests	1.25	0.78
Mean turnaround time	0.39	0.20
Likelihood of pathogen detection	1.40	1.61
Multiple-pathogen detection	1.07	2.07

Abbreviation: PCR, polymerase chain reaction.

To better characterize the need for testing, providers should prioritize the use of specific pathogen-related *ICD-10* codes (eg, *E coli*, *Shigella*, or rotavirus) when available and applicable, rather than relying on broad, nonspecific codes such as A09 (“Diarrhea of presumed infectious origin”). Coding must align with necessity as documented in clinical notes, such as prolonged symptoms lasting >7 days, high-risk conditions (eg, immunosuppression), or severe gastrointestinal illness. Furthermore, using additional codes for relevant symptoms (eg, R19.7, diarrhea unspecified; R50.9, fever unspecified) or outbreak-related exposure (eg, Z20.828, contact with viral infections) can help to justify the need for panel testing. Providers must also ensure that *ICD-10* codes adhere to the coverage criteria outlined in payor-specific LCDs and associated billing and coding articles, with claims supported by thorough documentation and aligned with established guidelines.

Since *ICD-10* lacks codes that explicitly capture the severity or duration of symptoms, an update to the coding system to address these factors would significantly enhance its utility. Current *ICD-10* codes primarily classify symptoms (eg, diarrhea, vomiting, fever) or specific pathogens but fail to reflect important clinical elements, such as chronicity, intensity, and impact on patient management, each of which is closely tied to medical necessity for advanced testing or treatment. For example, reimbursement for expanded gastrointestinal panels often hinges on factors such as prolonged symptoms (>7 days), recurrent infections, or severe illness, but these elements are typically documented in clinical notes rather than encoded in the *ICD-10* system. Incorporating codes that specifically represent duration (eg, prolonged diarrhea) or codes for grading the severity of symptoms (eg, severe diarrhea) could help highlight the medical necessity. Such updates would reduce ambiguity, streamline claims processing, and allow providers to better justify advanced diagnostics.

Furthermore, guidelines should be updated to explicitly define the clinical indications for testing and the rationale for utilizing molecular syndromic panels. Current guidelines, including the 2017 IDSA and 2016 American College of Gastroenterology [78, 79] guidelines, provide a solid framework but should be expanded to address the evolving role of advanced multiplex testing in clinical practice [81]. Updates could specify scenarios in which syndromic panels are appropriate, as in patients with severe or prolonged symptoms, individuals who are immunocompromised, suspected outbreaks of infectious gastroenteritis, and cases in which rapid diagnosis is critical for guiding therapy and preventing transmission. Furthermore, the guidelines should address the clinical benefits of syndromic panels for detecting coinfections or rare pathogens that are missed by conventional diagnostic methods. Emphasizing the role of multiplex panels in improving patient outcomes, antibiotic stewardship, and public health surveillance would support the alignment of payor reimbursement policies with clinical practice.

In lieu of arbitrary restrictions on panel size, payors are encouraged to reimburse expanded panels so that diagnostic decisions are driven by clinical necessity rather than cost containment, as missed diagnoses can lead to inappropriate therapy and greater downstream costs. Health care systems should also actively seek reimbursement for patients who meet established LCD criteria, as panels aligned with documented medical necessity provide critical information for patient management. Collaboration between payors and health care providers to align reimbursement practices with clinical guidelines would promote more equitable access to advanced diagnostic tools and reduce administrative burdens, enabling better outcomes for patients and health care systems.

A large study demonstrated the cost-effectiveness of multiplex PCR panels for gastrointestinal infections, showing that they reduce the need for additional diagnostic tests and unnecessary antibiotic use [77]. By providing faster, more accurate pathogen detection, large multiplex panels are associated with shorter hospital stays as compared with conventional testing methods, resulting in overall cost savings [51]. Moreover, larger panels have been associated with lower 30-day acute gastroenteritis–related follow-up costs and a decreased risk of acute gastroenteritis–related hospitalizations when compared with smaller panels [77].

Finally, laboratories that selectively report the results from multiplex PCR panels should reconsider this practice, as the reporting of all relevant detections can enhance clinical decision making and patient outcomes. For example, releasing *C difficile* results for adult patients with community-onset colitis of unknown etiology who lack specific risk factors for CDI can improve patient outcomes and reserve targeted *C difficile* PCR for patients with CDI risk factors or hospital-onset diarrhea [86]. A recent study found that *C difficile* is presently the most commonly detected pathogen in the community setting [2]. Community-onset CDI is often unsuspected and requires careful clinical assessment to confirm the diagnosis. Unlike other bacterial causes of acute gastroenteritis, CDI necessitates targeted treatment strategies, emphasizing the importance of its accurate identification and management in outpatient care settings. Similarly, reporting detections of EPEC or EAEC may be particularly beneficial for patients with persistent diarrhea, as these findings could support a trial of antibiotic therapy. The interpretation of these results should remain at the discretion of the clinical provider, and laboratories are advised to report results in full to ensure that providers have the complete information necessary for optimal patient care. Concerns regarding potential overtreatment of colonization or misinterpretation of results can be best alleviated by education and guidance to reinforce best practices rather than masking of results.

## CONCLUSIONS

Multiplex PCR panels have transformed the diagnosis of acute gastrointestinal infections, offering superior diagnostic accuracy and higher pathogen detection rates than conventional methods. While their initial reagent costs are higher than traditional testing methods, these costs are offset by lower overall health care expenditures. More rapid and accurate diagnostic testing without a net increase in health care costs should be welcomed by patients, providers, institutions, payors, and public health workers alike. Yet despite these well-documented benefits, inconsistent regulatory guidance, payor coverage, and laboratory implementation are preventing the full realization of the benefits of this advance in diagnostics. Rectifying this situation will require the harmonization of LCD coverage criteria, updating of *ICD-10* codes for acute gastroenteritis to align with reimbursement requirements, and refinement of clinical guidelines to clearly define the conditions that warrant multiplex PCR testing. These measures will facilitate successful reimbursement, promote equitable access to advanced diagnostics, and ultimately improve patient outcomes.
